# Marine eukaryote bioluminescence: a review of species and their functional biology

**DOI:** 10.1007/s42995-024-00250-0

**Published:** 2024-09-20

**Authors:** Laurent Duchatelet, Sam Dupont

**Affiliations:** 1 Marine Biology Laboratory, Earth and Life Institute, Université Catholique de Louvain, UCLouvain, Louvain-la-Neuve, Belgium; 2 Department of Biological and Environmental Sciences, University of Gothenburg, Fiskebäckskil, Sweden; 3 International Atomic Energy Agency Marine Environment Laboratories, Radioecology Laboratory, Principality of Monaco, Monaco

**Keywords:** Luminescence, Diversity, Taxonomy, Wavelength, Bioluminescence system, Bioluminescence functions

## Abstract

Bioluminescence, the ability of organisms to produce visible light, has intrigued scientists for centuries. Studies have examined bioluminescence, using a wide range of approaches and organisms, from its ecological role to its underlying molecular mechanisms, leading to various applications and even a Nobel prize. Over the last ten years, an increasing amount of data has been collected leading to a growing number of recognized marine bioluminescent species. This review provides and describes a referenced listing of the eukaryotic luminous marine species, including information related to: (i) intrinsic versus extrinsic source of the bioluminescence, (ii) the color and maximum wavelength of emission, (iii) the bioluminescent system (substrate and enzyme) and the associated molecules, (iv) the availability of light organ/cell(s) pattern and histological structure, (v) the physiological control of the light production, and (vi) the demonstrated or suggested bioluminescent function(s). This listing provides basic information and references for researchers in or entering in the field of marine bioluminescence. Using a semi-quantitative approach, we then highlight major research gaps and opportunities and reflect on the future of the field.

**Supplementary Information:**

The online version contains supplementary material available at 10.1007/s42995-024-00250-0.

## History of marine bioluminescence studies

**Fig. 1:**
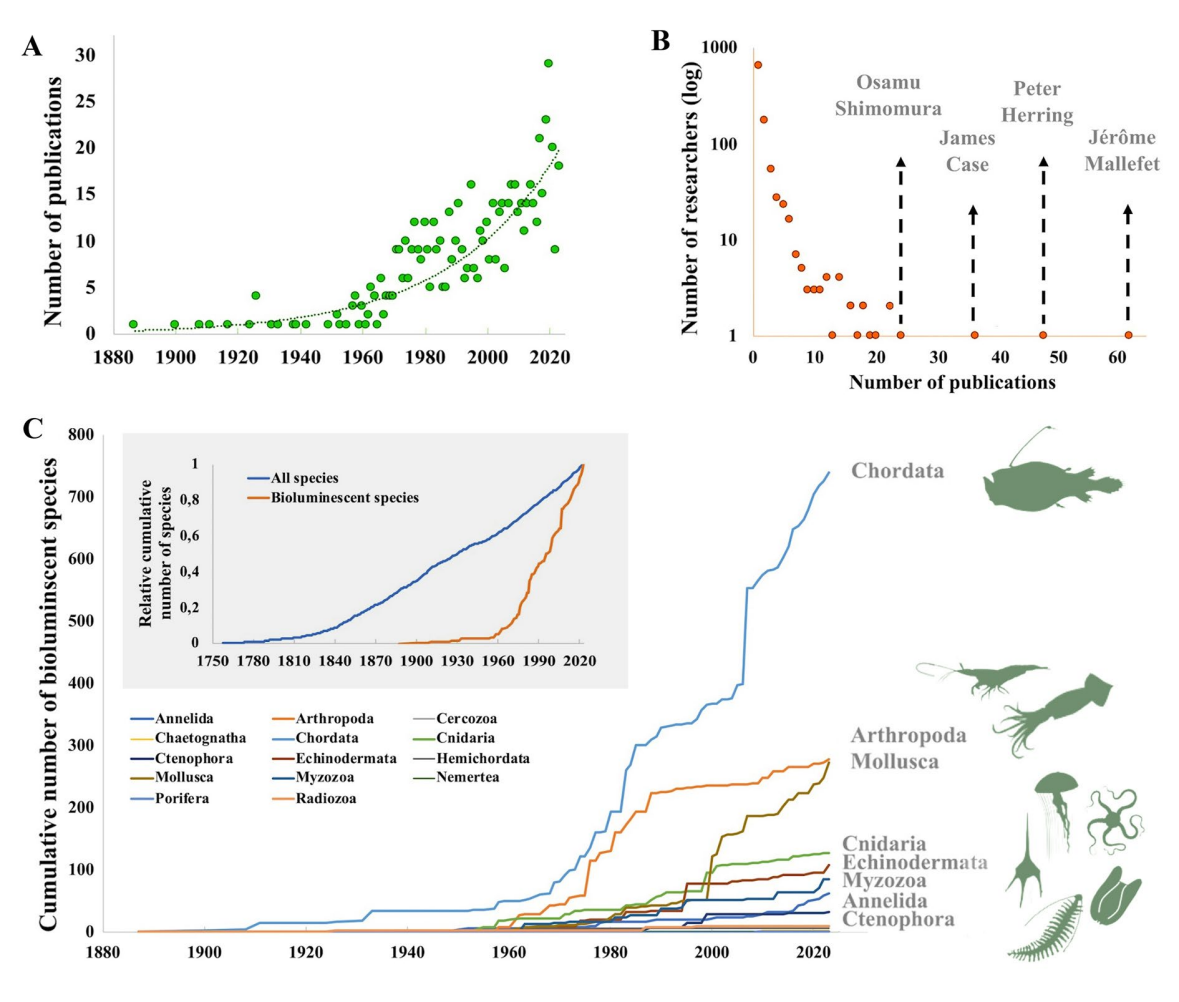
Evolution of bioluminescence studies. A Number of publications reporting bioluminescent marine eukaryote species over time. B Cumulative number of bioluminescent species over time per eukaryote phylum. Major bioluminescent taxa are schematized. Top left inset: the relative (i.e., scaled to 2023) cumulative number of marine bioluminescent and all animal species over time. See Table S1 for raw data. C Contribution (in log_10_ number of publications per author) of researchers involved in the study of bioluminescent marine eukaryote species. The four most productive researchers are highlighted

Bioluminescence, light production by a living organism, is a phenomenon that has attracted interest for more than 3000 years (Lee 2008), with records of descriptions and studies dating back to antiquity. For example, Aristotle mentioned in his book “*De Anima*” a “cold light” coming from the ocean. Many centuries later, while on board the H.M.S. Beagle, Charles Darwin noted a “milky sea” in his logbook. The first studies on the mechanisms underlying this phenomenon were in 1667 by Robert Boyle, who described the oxygen requirement for luminescence production (Shimomura 2012). In 1887, Dubois extracted the two main compounds of the luminescence chemical reaction, luciferin and luciferase (Harvey 1957). Today, the study of bioluminescence is an established area of scientific studies taking advantage of new techniques and technologies. Researchers have even detected bioluminescence from space via satellite sensor systems (Miller et al. 2005). Over the last few centuries, a large body of evidence has documented various aspects of bioluminescence. Harvey and Nicol were among the first to examine bioluminescence, from the morphology of luminous organs and cells to its physiological control (e.g., Harvey 1941, 1956; Nicol 1958). Other scientists left their mark in the field (Fig. 1C; e.g., on the diversity, bioluminescence physiological control, bioluminescence system analyses, general ecology, luminous behaviors, light emission patterns). Shimomura is one of the best-known researchers in this field and was awarded the Nobel Prize for Chemistry in 2008 following his discovery and description of the green fluorescent protein associated with the luminous system of the Medusozoa, *Aequorea* (Shimomura 1995; Tsuji 2010). Several excellent reviews have been published, summarizing the advancement of the field (e.g., Anctil 1979, 2018; Bessho-Uehara et al. 2024; Duchatelet et al. 2021; Haddock et al. 2010; Hastings 1968, 1996; Herring 1977, 2007; Mallefet 2009; Morin 1983; Shimomura 2012, 1983; Widder 2010).

Despite progress over the last decades, bioluminescence remains a mysterious phenomenon with many open questions regarding its mechanisms (e.g., light emission wavelengths, bioluminescence systems and associated molecular actors, physiological control, luminescence ecological role), ecological role and evolutionary history (Table S1). The evolutionary origin of bioluminescence also remains enigmatic, with two hypotheses currently proposed (Haddock et al. 2010; Widder 2010). One suggests a substrate-based origin, as most of the luciferins display antioxidant properties and might have originally served as defensive pathways against reactive oxygen species. These proteins may have been turned into light production compounds in environments with reduced oxidative stress (Rees et al. 1998). The second hypothesis suggests that the enzyme mutated and switched its intracellular role from its original function as an oxygenase to a role as a light production catalysis (McCapra 1990). Bioluminescence has probably evolved independently more than 90 times (Davis et al. 2016; Hastings 1983, 1995; Lau and Oakley 2021), suggesting that it plays important ecological roles and that the acquisition of light emission is a relatively easy and quick process (Haddock et al. 2010). This is an example of evolutionary convergence with no single common ancestor to all bioluminescent species. To illustrate this complex evolutionary diversity, Harvey wrote: “It is as if the various groups had been written on a blackboard and a handful of damp sand cast over the names. Where each grain of sand strikes, a luminous species appears” (Harvey 1952). The phylogenetic distribution of luminous species is paired with the diversity of ecosystems where luminous organisms live. Bioluminescence is present from marine to terrestrial ecosystems and from the poles to the tropics. Across luminous taxa, 80% inhabit marine environments from the coastal shallow waters to the abyssal deep-sea plains (Haddock et al. 2010; Morin 1983; Widder 2010). The terrestrial bioluminescence is restricted to a few taxa such as bacteria, fungi, annelids, mollusks, and arthropods (Oba et al. 2011; Shimomura 2012; Widder 2001). Only one bioluminescent species has been identified in freshwater, a limpet endemic to New Zealand (Kaskova et al. 2016; Shimomura 2012). The disparity in this distribution and the greater proportion of bioluminescent marine organisms could be explained by changes in abiotic factors, principally fluctuations in oxygen levels in the oceans over time (e.g., great oxidation event starting Paleozoic) (Reinhard and Planavsky 2022). Changes in abiotic conditions in the oceans are slow and generally last longer than in terrestrial environments, potentially leading organisms to adapt and fix more permanently newly evolved mechanisms to face modified conditions. Considering the putative antioxidant origin of the bioluminescence, organisms faced with an increase in oxygen levels and reactive oxygen species, would have evolved physiological enzymatic defense mechanisms, one of which being bioluminescence.

Currently, our understanding of bioluminescence remains hindered by lack of a compendium and evaluation of the wide array of marine eukaryotic bioluminescent species. To fill this gap, we performed a literature search on such species. Only taxa that were confirmed to be bioluminescent were considered. Species assumed to be bioluminescent (see *Methods*) were excluded. In doing so we provide information related to: (i) intrinsic versus extrinsic source of the bioluminescence, (ii) the color and maximum wavelength of emission, (iii) the bioluminescent system (substrate and enzyme) and the associated molecules, (iv) the availability of light organ/cell(s) pattern and histological structure, (v) the physiological control of the light production, and (vi) the demonstrated or suggested bioluminescent function(s). Using a semi-quantitative approach, we then highlight major research gaps and opportunities and reflect on the future of the field. The generated database provides a resource for scientists working on or starting in the field of marine bioluminescence research.

## Methods

The literature was screened through online databases (Google Scholar, ResearchGate, Web of Science, between April and December 2023). A first screening was made using the keywords: “bioluminescence”, “luminescence”, “luminous”, “photophore(s)”, “photocyte”, “luciferin”, “luciferase”, “photoprotein”, “color”, “wavelength”, “control”, “function”, “counterillumination”, “burglar alarm”, “startle effect”, “smoke screen”, and “lure”, alone or in combination. A second screening was performed through taxonomic entry (e.g., by phylum, family, or species) associated with the above keywords. Further references were identified using studies that were cited by sources obtained through the literature search. Abstracts were first screened for relevance, and then information was extracted from the full articles. Personal communications, articles that lacked clear taxonomic identification, and articles in which the species name could not be validated (Worms website https://www.marinespecies.org/) were excluded. Moreover, the organism had to be determined to the species level; taxa considered at higher taxonomic level (e.g., genus) were excluded as not all species within a genus are bioluminescent (e.g., *Amphiura filiformis* is bioluminescent while, *A. chiaje* is not; Mallefet et al. 2020). We did not include species that were considered bioluminescent based on weak or unvalidated evidence (i.e., mentioned without evidence of bioluminescent or species based on poor or incorrect taxonomic identification). As an example, the dinoflagellates *Gymnodinium flavum*, *Akashiwo sanguinea,* and *Prorocentrum micans* have been repeatedly mentioned in the literature as luminous (e.g., Santhanam 2022), while other studies invalidate this claim (Valiadi et al. 2012). Similarly, we did not include species hypothesized to be luminous (e.g., all Etmopteridae; Duchatelet et al. 2021) if no evidence was provided to support the claim (i.e., luminescence observations, light emission measurements, or photogenic structure morphology).

A total of 686 articles were selected and used to build a database of 1718 luminous species (Table S1). When available, the database also included: meta-data on (i) the intrinsic (i.e., own light production) versus extrinsic (i.e., provided by symbiotic luminescent bacteria) source of the bioluminescence; (ii) the color and maximum wavelength of emission; (iii) the biochemistry of the light production (substrate, enzyme, and associated actors); (iv) the photogenic structure pattern and/or morphology and histology; (v) the physiological control (nervous or hormonal as well as the effectors responsible for the light emission control); and (vi) the suggested or demonstrated ecological bioluminescent function(s). Furthermore, for each article, the name of each author was extracted, independently from the authoring position. A further database was created compiling the number of individual publications per author.

### Database general description

Since the first publication by von Lendenfeld (1887), 686 publications have documented the bioluminescence of marine eukaryote species. The number of publications per year appears to increase at an increasing rate, suggesting an exponential-like interest in the topic since 1887, reaching about 20–25 articles per year in the 2020s (Fig. 1A). However, by examining the position of data points relative to our fit of an exponential function (i.e., the residuals), it appears that around 1960, there was a change in the rate of publications, with an increase in rate after 1960 (Fig. 1A).

This shift in the 1960s is also reflected in the relative cumulative number of species identified as being bioluminescent (Fig. 1B). To date, 1718 bioluminescent species of marine eukaryotes have been identified. While fewer than 100 species were discovered between the first modern study in 1887 (von Lendenfeld 1887) and 1960. In 1960, the area of research increased, with a rate of discovery of ~ 27 new species per year between 1960 and 2023 (linear regression between the cumulative number of species and time; R^2^ = 0.99, F_1,60_ = 5569.64, p < 0.0001). We have compared the relative cumulative rate of discovery of new bioluminescent marine animal species with the relative cumulative rate of discovery of all new marine animal species as extracted from the Worms website on January 11, 2024 (Fig. 1C). Over the period 1960 to 2023, the number of new species increased linearly for both groups, but the rate of discovery was 2.6 times higher for bioluminescent species (relative rate of discovery, calculated relative to the total number of species over that period of time and ranging between 0 and 1, of 0.0156 per year, R^2^ = 0.99, F_1,60_ = 5570, p < 0.0001) as compared to all newly discovered species (relative rate of discovery of 0.0061 per year, R^2^ = 0.99, F_1,64_ = 49,143, p < 0.0001).

A total of 987 authors contributed to these publications but two-thirds (650 authors) only contributed to one publication (Fig. 1C). While this analysis does not reflect the relative contribution of authors or changes in publication practices over time (e.g., number of authors or position of authors name), it illustrates the establishment of bioluminescence as a standalone field of research with some researchers building their careers and laboratory focusing mostly on the subject. We suggest that our database could now be used for more in-depth bibliographic analyses and test hypotheses on the evolution of science publication practices.

Collectively, the increase in described species and publications (Fig. 1A, B), especially since the 1960s, seems to reflect an increased interest in the field of marine bioluminescence. Fully interpreting this observation is beyond the goal of this review. However, we can speculate that it is a consequence of the increasing interest of ecological research into ocean life. In the 1960s and 1970s, an increase in research occurred at institutions around the world (e.g., Station Biologique de Roscoff, Woods Hole Oceanographic Institute; Gage and Tyler 1991; Maienschein 1989). Their findings of unexpectedly high species diversity in the deep-sea—previously thought to be unhabitable—inspired marine ecologists (Gage and Tyler 1991). With the first aims being to theorize how high diversity could be maintained in such a poor and seemingly hostile environment, researchers focused on the species inhabiting the deep sea, notably luminous organisms. A seminal article by Harvey (1957) provided the first detailed review on all forms of luminescence, undoubtedly stimulating work in the 1960s and beyond. Certainly, technical advances also contributed. For example, the Trieste bathyscaphe allowed dives to almost 11 km in the Marianna trench (Gage and Tyler 1991; Rozwadowski 2005; Walsh 1962), and multiple oceanographic campaigns then started to explore the deep-sea (e.g., Tektite I, II, American-built submersible Alvin) (e.g., High et al. 1973; Miller 1970). Furthermore, between 1950 and 1970, ocean exploration started to be publicized through the media, notably with Jacques Cousteau’s research, diving and publications, leading to a growing interest in and support for marine sciences, including bioluminescence. Recognizing this past history, we suggest that with new methodologies and approaches being developed (e.g., remotely operated underwater vehicles and robotics, improved sensors for luminometry and photography, molecular tools and associated software), we should now see a greater increase in the interest for marine eukaryote bioluminescence, akin to the shift in the 1960s.

The 1718 retrieved luminous species (Table S1) were not equally distributed between Kingdoms and Phyla (Table 1). No bioluminescent marine species were documented in two out of the four Kingdoms (Plantae and Fungi). Chromista has 10 phyla with marine species, with 3 containing bioluminescent species (0.46% of all the marine species of Chromista). The animal kingdom has 31 phyla with marine species, with 11 containing bioluminescent species (0.78% of all marine species of animals). The percentage of described marine eukaryote species differs between these phyla, vary between 0.01% (Porifera) and 17.11% (Ctenophora).

**Table 1:** Number of described marine species, marine bioluminescent species, and the percentage of bioluminescent species in the four eukaryote kingdoms and 14 phyla with bioluminescent species

Taxonomic level (Kingdom/*Phylum*)	Number of marine species (Worms database)	Number of marine bioluminescent species (This study)	Percentage of bioluminescent species
Plantae	10,495	0	0
(7 phyla)			
Fungi	1429	0	0
(5 phyla)			
Chromista	20,522	94	0.46
(10 phyla)			
* Mysozoa*	3126	82	2.62
* Cercozoa*	254	1	0.39
* Radiozoa*	497	9	1.81
Amimalia	209,141	1627	0.78
(31 phyla)			
* Annelida*	13,914	62	0.45
* Arthropoda*	59,167	278	0.47
* Chaetognata*	132	2	1.52
* Chordata*	23,994	739	3.08
* Cnidaria*	12,332	127	1.03
* Ctenophora*	187	32	17.11
* Echinodermata*	7573	108	1.43
* Hemichordata*	133	5	3.76
* Mollusca*	51,339	272	0.53
* Nemertea*	1333	1	0.08
* Porifera*	9343	1	0.01

**Fig. 2:**
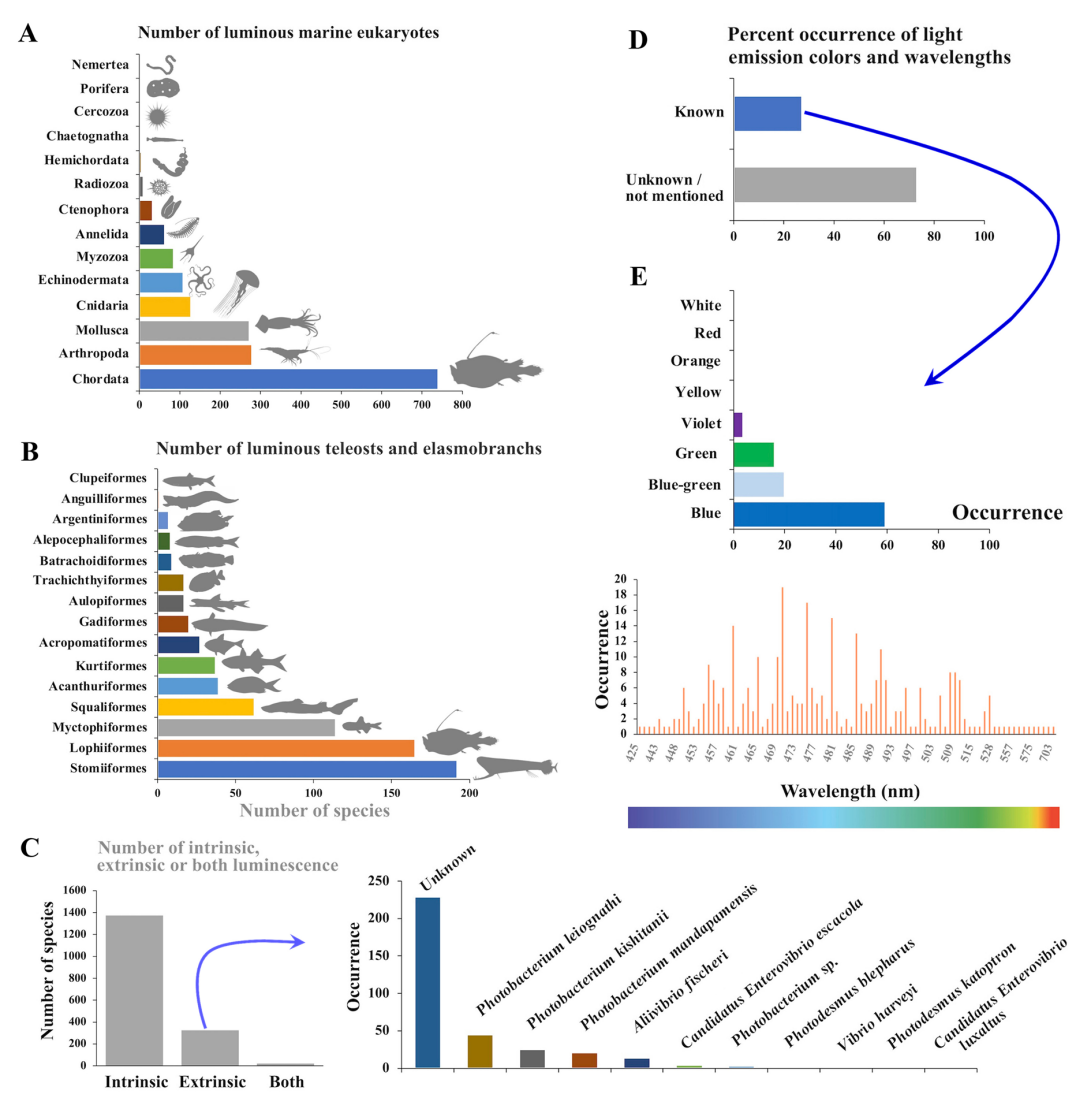
Summary of the data from the 1718 bioluminescent marine eukaryotes retrieved from the literature (Table S1). A Number of the luminous marine eukaryotes per phylum. B Number of the luminous teleosts and elasmobranchs per order. C Number of intrinsic, extrinsic, or both type of luminescence among the bioluminescent marine eukaryotes, with the occurrence of bacterial species encountered in extrinsic light emitters. D Percent occurrence of known and unknown light emission colors among the bioluminescent marine eukaryotes, with E percent occurrence of the known light emission colors and repartition of emission wavelengths

This translates into different number of bioluminescent species between phyla: 739 species (42.9% of bioluminescent eukaryotes) of chordate, 278 species (16.2%) of arthropods, 272 species (15.8%) of molluscs, 127 species (7.4%) of cnidarians, 108 species (6.3%) of echinoderms, 84 species (4.9%) of myzozoans, 62 species (3.6%) of annelids, 32 species (1.9%) of ctenophors, 9 species (0.5%) of radiozoans, 5 species (0.3%) of hemichordates, 2 species (0.1%) of chaetognaths, 1 species (> 0.05%) of nemertea, 1 species (> 0.05%), and 1 species (> 0.05%) cercozoans species (Fig. 2A). Within the chordates, bioluminescent species are encountered in urochordates (21 species), teleosts, and elasmobranchs (Table S1). Among the teleosts and elasmobranchs, 192 species (26.8%) are stomiiformes, 165 species (23.0%) lophiiformes, 114 species (15.9%) myctophiformes, 62 species (8.7%) squaliformes, 39 species (5.4%) acanthuriformes, and 37 species (5.2%) kurtiformes (Fig. 2B). The remaining species are represented respectively in less than 5% of the total number of bioluminescent species.

### Light emission types and colors

Among the 1718 described luminous species (Table S1) 80% are intrinsic emitters (i.e., species producing their own light), while 19% produce lights through an association with luminous symbiotic bacteria within morphological structures (Fig. 2C). In one instance (remaining 1%), the anglerfish genus *Linophryne* has both intrinsic and extrinsic luminescence (Hansen and Herring 1977). For extrinsic emitters, the associated luminous bacteria species are presented in Fig. 2C, although for the majority (66%) the bacterial symbiont are unknown or undetermined (Fig. 2C).

The color of the bioluminescence was mentioned for 462 species, and the maximum emission wavelength (λmax) was measured for 339 species (Fig. 2D; Table S1 Bioluminescence color, Wavelength). Previous reviews (e.g., Latz et al. 1988; Widder et al. 1983, 2010) indicate that blue/blue green emission is the main light, followed by green and violet. Other colors (white, yellow, orange, red) are less frequent. Our analysis supports this trend, although we have made finer distinctions between color categories (Fig. 2D). From our database, the division of colors is: 59.1% of blue, 19.8% of blue green, 16% of green, 3.6% of violet emitters. Other colors (yellow, orange, red, white) represent less than 0.45% each. The monochromatic light spectra of a specific photogenic source can vary between species, ranging from 408 and 425 nm for the teleost *Searsia koefoedi* and the cephalopod *Chtenopteryx sicula* to 702 and 703 nm in the dragonfishes *Malacosteus niger* and *Aristostomias scintillans* (Herring 1983; Latz et al. 1988; Widder et al. 1983, 1984). In general, the emission wavelength depends upon the environment the luminous organism inhabits. Most terrestrial bioluminescent organisms emit a yellow-green light, while coastal species are mostly green emitters, and pelagic and deep-sea species emit blue (Herring 1983)—water is an effective color filter and only blue light remains at few hundred meters (Young 1983). A larger diversity of wavelengths is observed in coastal waters. It was hypothesized that luminous organisms tend to match the light in their environmental (Haddock et al. 2010; Widder et al. 2010; Young 1983). It should be noted that species such as *Malacosteus niger*, *Aristostomias scintillans*, *A. tittmanni*, and *Pachystomias microdon* are able to emit light in two distinct wavelengths (i.e., blue and red) via morphologically distinct light organs (i.e., photophore) localized in different part of the body (i.e., ventral photophore and orbital/suborbital photophores). These serve different functions: (i) ventrally, the blue light serves for counterillumination (see *Bioluminescence functions*), while the orbital/suborbital red luminescence helps during predation by illuminating prey with a singular wavelength for deep-sea organisms (far-red light coupled with red visual pigment) (Douglas et al. 2000, 2002; Herring and Cope 2005).

The trends revealed by this review support previous hypotheses (e.g., match between emission spectra and light in the environment). Future work should focus on testing these by taking advantages of our database to generate hypotheses and technological advances in the field to make in situ observations as well as functional and behavioral laboratory-based experiments.

### Biochemistry of light emission

Light emission results from the biochemical reaction occurring within the light organ/cells; this involves oxidation of a substrate (i.e., luciferin) through the catalytic action of an enzyme (i.e., luciferase), and electronically excited oxyluciferin, which then releases photons as it relaxes to the ground state (Shimomura 2012). This general reaction is ubiquitous across luminous organisms. In some species such as *Mnemiopsis leidyi*, *Bolinopsis infundibulum*, *Beroe abyssicola*, *Clytia hemisphaerica*, *Obelia geniculata, Mitrocoma cellularia*, *Aequorea victoria*, *Malgremia lunulata*, *Pholas dactylus*, and *Stenoteuthis oualaniensis*, luciferase and luciferin are associated in a single complex called photoprotein, requiring additional co-factors to be functional (Shimomura 2012). The generated light color depends on the types of luciferins and luciferases, such as their amino acid sequences and structures (Shimomura 2012). For some species, the light emitted results from an interaction between luciferase and a fluorescent protein. The latter absorbs the photons emitted and enters an excited state. It then re-emits the light but converts it to longer wavelengths, returning to its ground state. This intracellular color change occurs, for example, with the green fluorescent protein (GFP), which re-emits green light after exposure to the initially blue light emitted by the classic luciferin-luciferase reaction (Loening et al. 2007; Shimomura 2012; Ward and Cormier 1979).

**Fig. 3:**
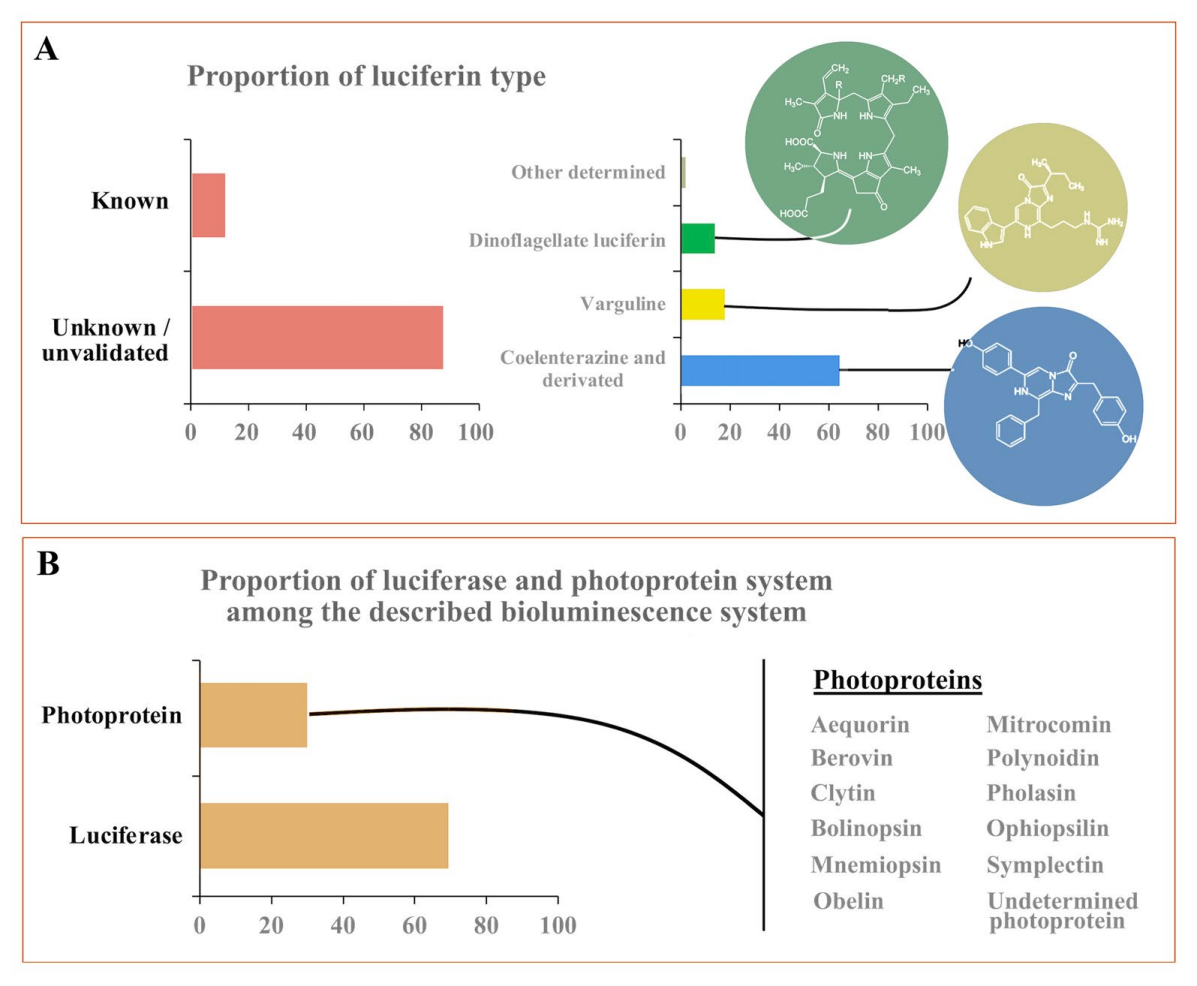
Summary of the bioluminescence systems from the 1718 bioluminescent marine eukaryotes retrieved from the literature (Table S1). A Percent occurrence of luciferin types in intrinsic bioluminescent marine eukayotes. B Percent occurrence of luciferase and photoprotein systems among the described bioluminescent systems, with a list of known photoproteins

Of the 1398 species using an intrinsic luminous system, luciferin has been identified from only 169 (12%) of these (Fig. 3A; Table S1 Bioluminescent substrate). Within these, three main luciferins have been observed: coelenterazine, vargulin, and dinoflagellate luciferin (Fig. 3A). A total of 65% use the coelenterazine or a derivative (i.e., dehydrocoelenterazine, coelenterazine disulfate), while 18 and 14% use the vargulin and the dinoflagellate luciferin as the substrate for the light production, respectively. This large occurrence of coelenterazine across many taxonomic levels suggests that it may be acquired by trophic transfer rather than intrinsic production (Coubris et al. 2024a, b; Duchatelet et al. 2019a; Frank et al. 1984; Haddock et al. 2001; Mallefet and Shimomura 1995; Mallefet et al. 2020; Thomson et al. 1997). For intrinsic light emitters, for which the bioluminescent system has been described, photoproteins are encountered in 30% of the species, while the luciferase-dependent system was observed in 69.6% (Fig. 3B; Table S1 Bioluminescent enzyme, Sequences).

While our knowledge on the biochemistry of light emission is improving, our data base has revealed that we only have data for a limited proportion of the known bioluminescence species. Expanding this approach to more species will help us appreciate the evolutionary and physiological implications of this phenomenon.

### Bioluminescent structures

Bioluminescent reactions, either from luciferin/luciferase or photoprotein systems, occur in a variety of structures. The simplest structural units involved in the light emission are specific organelles, called microsources, found in the photocyte (i.e., the luminous cell). Microsources bear different names according to the phylogenetic affiliation of the organisms in which they are present; i.e., scintillons: dinoflagellates; lumisomes: coelenterates; photosomes: polychaetes; membrane network: crustaceans; vesicles: brittlestars; glowons: sharks; microvesicles: fish (Anderson and Cormier 1973; Bassot and Bilbaut 1977; De Sa et al. 1963; Deheyn et al. 2000; Duchatelet et al. 2023b; Herring 1985b; Renwart et al. 2015). Luminescent unicellular eukaryotes have organelles for light production (Sweeney 1980). Single photocytes, not clustered to form an organ and without auxiliary structures, are found in comb jellies and some echinoderms. In several luminous organisms, photocytes are grouped together, enclosed and coupled with other structural elements, to form a light-emitting organ, the photophore (Herring 1985a). The majority of luminous metazoa harbors several to billions of photophores, each displaying complex photogenic structures (Duchatelet et al. 2021; Haddock et al. 2010; Herring 2000; Munk 1999; Paitio and Oba 2024; Sweeney 1980). These additional complex structures of the photophore increase light emission efficiency, such as reflectors directing light, lenses and light guides focusing the light, filters tuning the light emission wavelength (Denton et al. 1972, 1985; Duchatelet et al. 2020a, b, c; Herring 1985a, 2000; Paitio and Oba 2021). Additionally, pigmented cells may act as shutter regulating the amount of light emitted (Duchatelet et al. 2020a, b, c; Paitio and Oba 2021). Other structures may help to physically control the light emission such as a muscularly controlled membranes, which cover the photophore (Haygood 1993; Howland et al. 1992). Photophores may contain luminous secretion, which either are released externally (i.e., extra-glandular or secretory luminescence) or remain within the organ structures (i.e., intra-glandular luminescence) (Herring 1985a; Herring and Morin 1978; Shimomura et al. 1978). Photophores may also contain symbiotic luminous bacteria (extrinsic luminescent system). While the host organism takes advantage of the bacterial light emitted for various purposes (see *Bioluminescence functions*), symbiotic bacteria are provided with an adequate environment (e.g., shelter, oxygen, nutrient supply) to multiply within the host photophore (Dunlap and Kita-Tsukamoto 2006; Haygood 1993; Tanet et al. 2020). The photophore location varies between organisms, often reflecting the ecological function used (see *Bioluminescence functions*). Counterilluminating organisms mainly harbor ventral photophores to allow camouflage (Claes and Mallefet 2014; Herring 1985a; McAllister 1967; Young et al. 1979). In some case, this location is clearly related to the evolution history of the species. For instance, the internal symbiotic light organs of numerous fishes occur in and are derived from the intestinal tract (e.g., the genus *Coelorinchus*); likewise the squid light organ occurs in and is derived from the ink sac (e.g., *E. scolopes*) (Haygood 1993; Herring and Morin 1978; Tong et al. 2009).

The patterns and structures of light emission are used as criteria for taxonomic purposes. Information about these patterns, structures, and luminous organs exists for 480 of the 1721 species listed (Table S1 Structure description). Among the studies presenting these data, only a few investigated the ultrastructure of these luminous organs. Most of these organs/cells are in close association with terminal nervous cell extensions. These observations have led scientists to investigate the physiological and neural control of light production, which we review in the next section.

### Bioluminescence controls

To efficiently fulfill its ecological function (see *Bioluminescence functions*), bioluminescence must be finely controlled at the physiological level. The control of bioluminescence includes: (i) physiological control of bacterial luminescence, (ii) biophysical control in unicellular eukaryotes, and (iii) neuronal and hormonal control of the light organ in Metazoa (Case and Strause 1978; Claes and Mallefet 2009; Duchatelet et al. 2021). However, these three mechanisms are rarely discrete. For instance, neural and physiological mechanisms (e.g., via neurotransmitters or hormones) can directly control the photocytes, but they may also control light emission by directly regulating structural photophore elements (e.g., optical filters, lenses, chromatophores) or indirectly regulating elements linked to photophores (e.g., muscles). Therefore, clear distinction between mechanical controls needs to be taken with caution. Below we expand on these mechanisms.

Physiological and biophysical control mechanisms are particularly developed in extrinsic light-emitters. Since bacteria emit light continuously and reach a stable population within the extrinsic photophore, hosts need to develop control mechanisms to regulate the light emitted and manage the bacteria population growth (Haddock et al. 2010; Haygood 1993). Bacteria regulate their luminescence upon a population density-dependent mechanism (i.e., quorum sensing) in which the population needs to reach a certain threshold to emit light (Case and Strause 1978; Hastings 1978; Haygood 1993). Bacteria continuously release an autoinducer compound in the environment that specifically binds to receptors from other bacteria, which then express the gene responsible for the bacterial light emission (i.e., the lux operon). Furthermore, bound autoinducers also up-regulate their own expression. This leads to a continuous increase of these autoinducers in the media in a bacteria density-dependent manner resulting in continuous glow (Dunlap and Kita-Tsukamoto 2006; Meighen 1993). Hence, there is a need for hosts to have structures or mechanisms to control this light emission. For instance, within flashlight fish, *Anomalops* species have light organs that are able to rotate into a subocular dark pocket, while *Photoblepharon* species have a mobile membrane that covers the subocular light organs; both these mechanisms allow luminescence to be switched on and off as “winks” (Herring 1982; Johnson and Rosenblatt 1988). Similarly, some anglerfish and the pinecone fish have light organs inside their mouths, which they open and close to regulate luminescence (Herring 1982; Karplus et al. 2014), while the leiognathid fish, *Gazza minuta*, use its opercula and branchiostegal rays to cover the gill chamber that contains a gland hosting luminous bacteria (McFall-Ngai and Dunlap 1983).

Biophysical mechanisms are seen in luminous dinoflagellates for which light production occurs through mechanical stimulation (e.g., flow gradient originating from waves, shear, predator swimming and swallowing) (e.g., Latz and Lee 1995; Latz and Rohr 1999; Latz et al. 2004; Marcincko et al. 2013; Vishal et al. 2021). Luminous dinoflagellates use specific G-protein-associated mechanoreceptors that trigger changes of pH upon plasma membrane deformation, resulting in light production (Chen et al. 2007). Briefly, upon activation, the mechanical signal is transmitted through a change of intracellular calcium concentration, triggering the propagation of an action potential along the vacuole membrane, that allows proton flux from the vacuole to the cytoplasm (von Dassow and Latz 2002; Eckert 1965; Rodriguez et al. 2017). The resultant pH change acidifies specific organelles, the scintillons, containing the dinoflagellate bioluminescent system, activating the luciferase that triggers light production (De Sa and Hastings 1968). Each dinoflagellate species possesses a specific shear threshold, which once reached, triggers the emission of light (e.g., Marcincko et al. 2013). Besides this mechanical stimulation response, dinoflagellates display variations in their bioluminescence depending on a diurnal rhythm, with a photoinhibition during the light phase and a high bioluminescence capacity and excitability during the dark phase (Biggley et al. 1969; Esaias et al. 1973; Fritz et al. 1990; Sweeney and Hastings 1957).

Direct neural regulation is mainly found in intrinsic luminescent organisms and has been extensively studied in Osteichthyes and Echinodermata. Among the first studied species, *Porichthys* have been demonstrated as a catecholamine-controlled light emitter (Anctil and Case 1976; Baguet and Case 1971; Christophe and Baguet 1983; Lariviére and Anctil 1986; Nicol 1957). Most of the nervous molecules involved in the control of light emission belong to three groups: (i) simple amino acids (e.g., g-aminobutyric acid (GABA), tryptamine, glutamate); (ii) classical neurotransmitters (e.g., serotonin, (nor)adrenaline, acetylcholine, purines, nitric oxide, octopamine); and (iii) neuropeptides (e.g., SALMFamide neuropeptides S1) (Nicholls 1994). Phylogenetic patterns exist regarding neural regulation. For instance, acetylcholine is the main neurotransmitter in Polychaeta and Ophiuroidea (Coubris et al. 2024a; De Bremaeker et al. 1996; Dewael and Mallefet 2002; Gouveneaux et al. 2013; Nicol 1952; Nicolas et al. 1978). The main neurotransmitter triggering luminescence of Anthozoa and Osteichthyes is adrenaline (Anctil et al. 1982; Baguet 1975; Duchatelet et al. 2023a; Mallefet et al. 2019). However, some closely related species do not share common nervous light emission control mechanisms (Dewael and Mallefet 2002). For instance, nitric oxide has a specific neuromodulator role in the neural-induced luminescence, as modulated light emission in species such as the krill *M. norvegica*, the midshipman fish *P. notatus*, the hatchetfish *Argyropelecus hemigymnus*, the pearlfish *Maurolicus muelleri*, and probably other fish species such as those within the Myctophidae (Krönström et al. 2005, 2007; Krönström and Mallefet 2010).

Another physiological control is unique to luminescent elasmobranchs. Rather than using neurotransmitters, they rely on hormones to regulate their light production (physiologically demonstrated in seven species); e.g., the hormone melatonin triggers light emission, while melanocortin inhibits light production (Duchatelet et al. 2021 for review).

Recently, a new type of control was proposed involving a photo-perception of the luminescence at the light production site (e.g., Bracken-Grissom et al. 2020; Duchatelet et al. 2020c; Tong et al. 2009). Several studies highlight through multiple approaches (e.g., transcriptomics, in situ hybridization, morphological analyses, immunohistochemistry) the expression of extraocular photoreceptors close to the photogenic sites. For instance, some deep-sea sharks have photoreceptors (i.e., opsin) that are involved in the perception of their own emissions. This mechanism allows them to discriminate between photophore emissions and background light and modify the photophore ultrastructure. Specifically, movements of pigments within the photophore can alter the intensity of the luminescence. Indeed, upon light absorption by extraocular opsin, expressed within the photophore, pigment movements have been described resulting in the modulation of the luminescence (Duchatelet et al. 2020c, 2021). Additional studies underlined the presence of photoreception proteins and ultrastructural photophore modification upon light absorption in deep-sea shrimp. This photoreception process is also assumed to be involved in accurate light emission for counterillumination (Bracken-Grissom et al. 2020).

Initial physiological studies on light emission control mainly demonstrated an electrophysiological dependence of luminescence by electric impulse or depolarization agent applications (e.g., Anctil 1987; Baguet 1975; Bowlby and Case 1991; Clarke et al. 1962; Cormier et al. 1974; Davenport and Nicol 1955; Davis and Conover 1961; Gouveneaux and Mallefet 2013; Harvey 1921; Herring 1981, 1991; Moore 1926; Nicol 1954, 1957; Nicolas et al. 1978; Rivers and Morin 2012; Satterlie et al. 1980; Zörner and Fischer 2007). Deeper investigations on the light emission control that examine the neuroeffectors, neurorepressors, and/or neuromodulators, and the underlying pathways are rare and on a few, select species (e.g., Baguet 1975; De Bremaeker et al. 1996, 1999, 2000; Dewael and Mallefet 2002; Doyle 1966; Duchatelet et al. 2020a,b,c, 2021; Dupont et al. 2004; Gouveneaux and Mallefet 2013; Herring and Locket 1978; Krönström et al 2007; Vanderlinden et al. 2010;). Mechanical or electrophysiological studies were performed on 166 species of the 1718 species we reviewed, and deeper investigation on the physiology and molecules involved in the light emission control were done on 74 species (Table S1 Mechanical/electrical/physiological stimulation, neuroeffector/neurorepressors/hormone). Physiological studies reveal that light production is triggered by serotonin (5-HT) in 15 species (12 of them being Malacostraca); acetylcholine in 15 species (mainly echinoderms and annelids); adrenaline in 15 species (mainly in cnidarian and teleosts); noradrenaline in 9 species (mainly cnidarian and teleost); and melatonin in 7 elasmobranchs species.

This review highlights the complexity and diversity of the physiological control of bioluminescence across marine Eukaryotes. We suggest that now this knowledge should be expanded to a wider range of species and phyla to provide a more systematic view and resolve the mechanisms as well as the ecological and evolutionary implications. This is an achievable goal thanks to the new tools available to physiologists as well as new technologies for sampling and maintaining bioluminescent species in the laboratory.

### Bioluminescence functions

Bioluminescence serves many functions for marine species and frequently fulfills multiple roles for a single organism (Haddock et al. 2010; Harvey 1952). However, bioluminescence is generally classified into three groups: defense against predation, attracting prey, and intraspecific communication (Buck 1978; Campbell 1989; Haddock et al. 2010; Hastings 1995; Morin 1983). Furthermore, bioluminescence varies in terms of light emission time, from rapid flashes (< 2 s) to long-lasting glows (> 5 s); long-lasting glows are thought to function as attractant signals and rapid flashes as repellent (Haddock et al. 2010; Morin 1983). Distance between organisms is also important, as a flash directed at short range may attract attention from afar (Haddock et al. 2010; Morin 1983). Below we evaluate these aspects, and others, in the context of the three main functions.

Defense is the most prevalent functional for bioluminescence, and this includes many mechanisms that allow prey to escape predators (Haddock et al. 2010). Whether through flashes or glows, organisms have evolved strategies to avoid predation such as a startle effect, luminous smokescreen, luring position, sacrificial tag, aposematic signal, counterillumination, or burglar alarm system (Buck 1978; Haddock et al. 2010). The function of bioluminescence has been suggested or demonstrated for 483 species, among which many functions have been proposed for the 107 species (Table S1 Bioluminescent function). Startle effect is suggested for 37 species; luminous smoke screen/cloud is suggested for 80 species; the use of sacrificial lure as a distractive flashing body part is suggested for 12 species (and demonstrated in one species); a sacrificial sticking luminous body part (i.e., during an interaction, luminous part of an organism that sticks to the primary predator’s body and can attract the attention of a secondary predator) is suggested for one species; aposematic use of luminescence is suggested for seven species (and experimentally demonstrated for one species); counterillumination (i.e., camouflage in which the organism emits light ventrally to cloak its silhouette when viewed from below) is the most suggested defensive function, with 136 occurrences; the burglar alarm strategy (i.e., light emission from a prey during predation event that attract the predator of the primary predator) is suggested for 14 species (and demonstrated for five other species).

Light can also be a powerful attractant. Therefore, numerous organisms have developed bioluminescent lures to attract prey and increase their predatory success. Bioluminescence can help predation by luring, stunning, and illuminating prey. Within our listing (Table S1 Bioluminescent function), an attractive lure is suggested to be used by 173 species (mainly teleosts); stunning preys is assumed for four species; and simple illumination strategy is hypothesized for 21 species.

Finally, bioluminescence can serve as an intraspecific communication channel allowing recognition for schooling, mating, or territorial signaling. Thirty-six species have been suggested to use intraspecific communication, although the purpose of the signal was not stated; light emission for schooling behavior is assumed for six species (and demonstrated for one species); the use of luminescence as courtship display for sexual recognition is suggested for 40 species (and demonstrated for 21); finally, for two species, luminescence is assumed to play a role in the territorial defense strategy against congeners (Table S1 Bioluminescent function).

As a final point, the function of bioluminescence in marine eukaryotes is often based on indirect or little to no evidence; a demonstration of a function requires ethological experiments, ideally in the natural environment (Campbell et al. 2009; Greene 2005; Werner 1992). Only a few studies adequately assess the ecological function of bioluminescence (e.g., flashes for reproduction for some ostracod species—Guerrish and Morin 2016; Rivers and Morin 2009, 2012; flashes production for defense against copepod grazing in dinoflagellates—Huang et al. 2023; Lindström et al. 2017; Prevett et al. 2019). Others often attribute a function based on morphology, emission wavelengths, lifestyles, or phylogenetic inferences (e.g., Buck 1978; Duchatelet et al. 2019b; Haddock et al. 2005, 2010; Kubodera et al. 2007; Mallefet et al. 2021; Robison 1992; Widder 1998). The difficulties in collecting, maintaining, and recording light emission events for most of marine organisms are the main barriers preventing scientists from developing behavioral studies. These challenges can be overcome thanks to the development of new technologies such as performant ultra-intensified high-resolution cameras, robotics and improved ROVs and submersibles, pressurized tanks, AI, and new bioinformatic software (e.g., Gruber et al. 2019; Hellinger et al. 2017, 2020; Jägers et al. 2021; Phillips et al. 2016). Despite a lack of direct evidence, bioluminescence may be important in structuring deep-sea ecosystems (Martini and Haddock 2017; Martini et al. 2019). A better understanding of the different uses of light production will improve our understanding of the functioning of these ecosystems.

## Conclusions and perspectives

The study of bioluminescence is a growing field of research as demonstrated by the exponential-like increase in the number of publications reporting bioluminescent species of marine eukaryotes as well as the large number of associated authors. Based on the current rate of discovery of new bioluminescent species, it is likely that many more bioluminescent species remain to be identified. While it is important for our understanding of bioluminescence to report new instances of bioluminescent species, it is equally important to report non-bioluminescent ones; specifically, those species that might be expected to be bioluminescent, e.g., based on taxonomic affinity to identified bioluminescent species. This can be facilitated by simplified methodologies (e.g., Standard Operating Procedures that will need to be developed and accepted by the community) and protocols that can be shared with taxonomists and ecologists.

In this semi-quantitative review, we have created a list of bioluminescence species of marine eukaryotes. This database, available as supplementary material, provides a resource for scientists working on or entering the field of marine bioluminescence studies. For example, it can be used to aid in choosing model organism(s) for a given question or to identify gaps in our knowledge on marine bioluminescence. These include questions regarding the mechanisms, evolution, and ecological role of light emission. There is also a high potential to discover new molecules with biotechnological applications. For example, the *Rluc* gene is one of the most used probes in biomolecular laboratories. This marine bioluminescent “pharmacopy” could lead to new advances in biomedical, bioimaging, molecular analyses, and many other applications (e.g., Belkin et al. 2017; Cevenini et al. 2015; Kassem et al. 2014; Kim et al. 2004; Love and Prescher 2020; Nakajima and Ohmiya 2010; Roda et al. 2004; Sharifian et al. 2017, 2018). This database will enable researchers to identify species for which data are lacking and could lead to the discoveries of new light emission mechanisms that can be used in biotechnology. Finally, bioluminescence can be used as a tool to answer classic scientific questions in the fields of ecology and evolution (e.g., functional ecology, convergent evolution), or even as a biomarker in the field of stress ecology (e.g., Hurtado-Gallego et al. 2019; Lau and Oakley 2021; Martini and Haddock 2017; Mashukova et al. 2023; Palani et al. 2022; Takenaka et al. 2017).

Our list is based on a literature review that extends to the end of 2023. New bioluminescent species are likely to be regularly described and the taxonomic and bioluminescent status of species described in this manuscript to evolve. We encourage the community to contact us to share new discovery, omissions, and expert evaluation of our list. We will keep updating and improve it and share the updated list upon request.

## Supplementary Information

Below is the link to the electronic supplementary material.

Supplementary file1 (XLSX 353 KB)

## Data Availability

All data supporting the findings of this study are available within the paper and its Supplementary Information.
